# Design of two-photon molecular tandem architectures for solar cells by *ab initio* theory†
†Electronic supplementary information (ESI) available: Visualizations of molecular orbitals, one-particle mechanisms and a table with Kohn–Sham eigenvalues. See DOI: 10.1039/c4sc03835e



**DOI:** 10.1039/c4sc03835e

**Published:** 2015-03-04

**Authors:** Kristian B. Ørnsø, Juan M. Garcia-Lastra, Gema De La Torre, F. J. Himpsel, Angel Rubio, Kristian S. Thygesen

**Affiliations:** a Center for Atomic-scale Materials Design , Department of Physics , Technical University of Denmark , 2800 Kgs. Lyngby , Denmark . Email: krbt@fysik.dtu.dk ; Email: thygesen@fysik.dtu.dk; b Department of Energy Conversion , Technical University of Denmark , Frederiksborgvej 399 , 4000 Roskilde , Denmark; c Departamento de Quimica Organica , Facultad de Ciencias , Universidad Autonoma de Madrid , Campus de Cantoblanco , 28049 Madrid , Spain; d Department of Physics , University of Wisconsin-Madison , 1150 University Avenue , Madison , Wisconsin 53706 , USA; e Max Planck Institute for the Structure and Dynamics of Matter , Hamburg , Germany; f Nano-Bio Spectroscopy Group and ETSF , Universidad del Pais Vasco CFM CSIC-UPV/EHU-MPC & DIPC , 20018 San Sebastian , Spain

## Abstract

An extensive database of spectroscopic properties of molecules from *ab initio* calculations is used to design molecular complexes for use in tandem solar cells that convert two photons into a single electron–hole pair, thereby increasing the output voltage while covering a wider spectral range. Three different architectures are considered: the first two involve a complex consisting of two dye molecules with appropriately matched frontier orbitals, connected by a molecular diode. Optimized combinations of dye molecules are determined by taking advantage of our computational database of the structural and energetic properties of several thousand porphyrin dyes. The third design is a molecular analogy of the intermediate band solar cell, and involves a single dye molecule with strong intersystem crossing to ensure a long lifetime of the intermediate state. Based on the calculated energy levels and molecular orbitals, energy diagrams are presented for the individual steps in the operation of such tandem solar cells. We find that theoretical open circuit voltages of up to 1.8 V can be achieved using these tandem designs. Questions about the practical implementation of prototypical devices, such as the synthesis of the tandem molecules and potential loss mechanisms, are addressed.

## Abstract 2

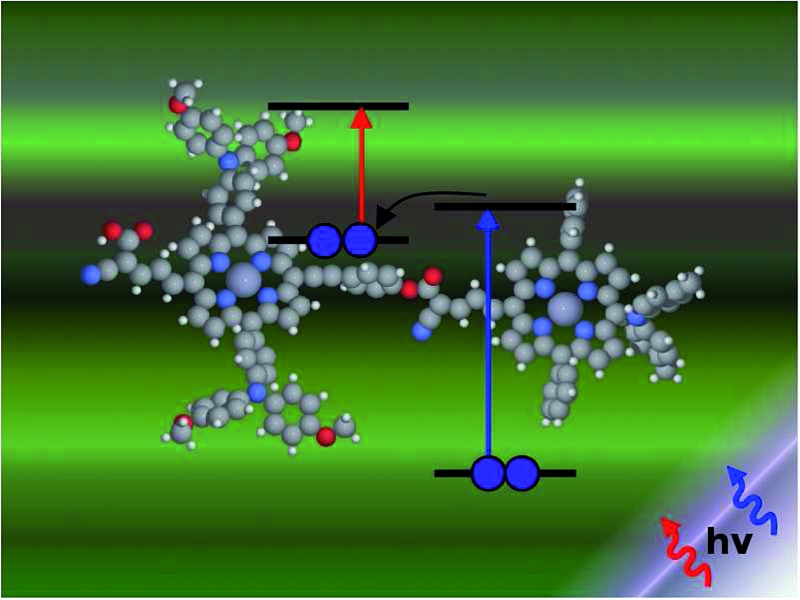
We present new two-photon molecular architectures for photovoltaics where atomic precision can be obtained by synthetic chemistry.

## 1 Introduction

As the search for renewable energy sources has intensified, the discovery of efficient and cheap technologies for exploiting the energy from the sunlight has emerged as a key challenge. Important examples of such technologies include organic polymer and small-molecule solar cells, as well as dye sensitized solar cells (DSSCs) which provide inexpensive, flexible, and environmentally friendly alternatives to the more conventional inorganic solar cells.^1–3^ One important drawback of the traditional molecular based photovoltaic systems is their inefficiency in capturing the red and infrared part of the solar spectrum. In fact, they are often transparent in the red, suggesting the addition of a second solar cell to intercept that part of the solar spectrum.^4^ Such tandem cells have the potential to double the output voltage of a solar cell at the expense of a lower current. As lower currents are associated with smaller losses and higher fill-factors, as observed in experiments under low light intensity,^5,6^ trading a high current for a high voltage could boost the efficiency of the cell.

Higher efficiency plays an increasingly important role in making competitive solar cell designs as the price of silicon continues to drop. At present, the actual silicon solar cell accounts for less than 1/4 of the cost of a complete solar panel installation and the cost of the support structure is becoming increasingly important. Higher efficiency reduces the required area and thereby reduces the cost of both the solar cells and their support structure.^7^ A way to increase the efficiency of a photovoltaic system is to incorporate the second solar cell into a tandem device. However, pn-type tandem DSSCs have not been able to surpass the efficiency of single DSSCs so far.^8,9^ Tandem designs have also been investigated for polymer solar cells^10,11^ (for further references on tandem designs in organic photovoltaics see ref. 51–58 in the review by Krebs^12^ and ref. 213–232 in the review by Cao and Xue^13^). Furthermore, dye sensitized upconversion has been suggested as a way to exploit infrared light.^14–16^

Conventional tandem solar cells (organic as well as inorganic) combine two or more types of materials that are separated by interfaces where electrons and holes are exchanged. These interfaces are highly critical and inevitably contain defects and other imperfections, which act as scattering and recombination centers for the charge carriers. The problem of controlling the atomic structure of the interface could be circumvented using molecular complexes where molecules absorbing in different parts of the solar spectrum are combined with atomic precision through synthetic chemistry. The design of such complexes is clearly a daunting experimental challenge as successful operation depends sensitively on the relative position of all the involved energy levels. However, using *ab initio* calculations it is now possible to search a large variety of possible materials and identify promising candidates which could be considered experimentally.^17–21^ Recently we have constructed a database containing calculations of the frontier orbitals of more than 5000 porphyrin dyes.^22,23^ Porphyrins have been widely used in DSSCs^24^ including the system with the highest reported efficiency so far.^25^ Here we take advantage of this database to propose new porphyrin-based molecular complexes inspired by the tandem^26^ and intermediate band^27–29^ solar cell schemes. The proposed molecular architectures have the potential to exploit a broader range of the solar spectrum and at the same time obtain very large open-circuit voltages. In addition, the proposed design combines the different photoactive regions through atomically well-defined chemical bonds and thereby eliminates the problems with disorder and defects at the interfaces in conventional tandem devices. We present our idea in the context of a DSSC and show that open-circuit voltages of up to 1.8 V are achievable in molecular complexes that generate a single electron–hole pair from two absorbed photons. Finally, we briefly discuss practical perspectives and challenges related to the realization of the proposed schemes.

## 2 Methods

The atomic and electronic structures of 5000+ porphyrins are taken from our public database^22,23^ (; http://cmr.fysik.dtu.dk) containing quantum mechanical calculations based on the use of density functional theory (DFT)^30^ with the PBE^31^ exchange–correlation functional as implemented in the GPAW code.^32^ The calculations use consistently a basis set of numerical atomic orbitals^33^ with double-*ζ* and polarization, a grid-spacing of 0.18 Å and a unit cell with a 5.0 Å vacuum added on all sides of the molecules. The structures have been optimized until all forces were below 0.05 eV Å^–1^. After the geometry optimization the location of the highest occupied molecular orbital (HOMO), *E*_HOMO_, and lowest unoccupied molecular orbital (LUMO), *E*_LUMO_, were calculated as the ionization potential *I*_P_ and electron affinity *E*_A_ of the molecule. Thus the resulting energy gap, *E*_gap_, is given by:

1 *E*_gap_ = *E*_LUMO_ – *E*_HOMO_ = (*E*[–1] – *E*[0]) – (*E*[0] – *E*[+1]) = *I*_P_ – *E*_A_

where *E*[0] is the ground state total energy and *E*[–1] and *E*[+1] is the total energy of the negatively and positively charged ions of the molecule in the ground state geometry, respectively. In the latter case the magnetic moment of the system is fixed to ensure a single unpaired electron. This definition of *E*_HOMO_ and *E*_LUMO_ avoids the use of Kohn–Sham (KS) eigenvalues which are well-known to be inaccurate within PBE. In addition we have previously shown that this definition gives good trends compared to experiments^22^ and we will therefore use *E*_HOMO_ and *E*_LUMO_ calculated from total energy calculations throughout this study. In addition to the fundamental gap, the lowest optical transition energy, *E*_1_, has also been calculated. The calculation of *E*_1_ is done by forcing the molecule to the triplet ground state by fixing the magnetic moment, and thus promoting one of the two electrons in the HOMO to the LUMO. We use the triplet excitation energy rather than the singlet excitation because this is technically simpler to compute. We have previously shown for a number of Zn porphyrins that the singlet and triplet excitations are within 0.3 eV and that their dependence on molecular structure is very similar.^22^ In the same study we furthermore showed that computed *E*_HOMO_ and *E*_1_ values compared well to experimental values.^22,34^ For selected dyes we have in this study calculated the singlet excitation energies using the all-electron ADF code with a double-*ζ* Slater-type basis set with polarization functions^35^ and the proposed dyad for the molecular tandem scheme has been investigated using TD-DFT as implemented in Orca^36^ with the CAM-B3LYP functional.^37^ Full details on the ADF and Orca calculations are given in the ESI.†

## 3 Molecular two-photon schemes

**Fig. 1:**
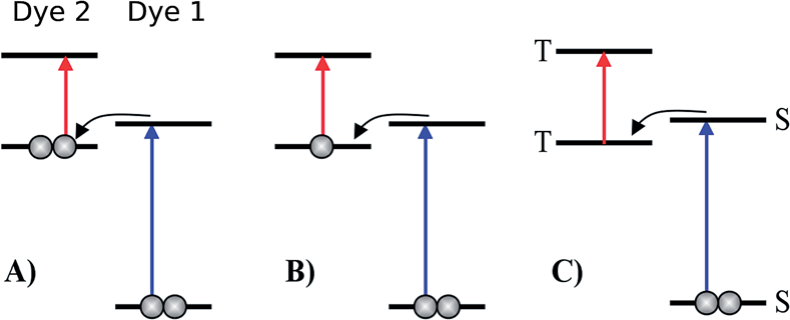
Various proposed energy level schemes for solar cells based on two dye molecules connected by a molecular diode (molecular linker not shown here, see Fig. 3). The occupancy is given for the ground state. (A) Simplest concept with both HOMOs doubly occupied. Both dye molecules need to be excited at the same time to generate a hole in the upper HOMO that can take up an electron from the lower LUMO. That is unlikely to happen in the same tandem complex. (B) The upper HOMO is partially occupied to allow facile charge transfer between the two dye molecules. Such a situation can be realized in metal–organic dye molecules with an odd number of electrons. This is the analog of an intermediate band solar cell.^27–29^ (C) Using singlet (S) and triplet (T) excitations. The long lifetime of the triplet allows a significant electron population to accumulate in the lower triplet level, which can be excited into the upper triplet level. This scheme could also be realized with a single dye molecule.

Inspired by earlier attempts to improve the light absorption in DSSCs by combining the conventional n-type DSSC with a p-type DSSC^38^ to construct a tandem pn-DSSC,^26^ the intermediate band solar cell design^27,28^ and especially the molecular version of this,^29^ as well as previous attempts to use supramolecular porphyrin structures to enhance the efficiency,^39–41^ we propose three different molecular two-photon schemes, shown in Fig. 1. The different schemes are explained in more detail in the following, where we also propose specific dyes as suitable candidates for experimental realizations of the different schemes.

### 3.1 Tandem scheme

The first concept we propose is the tandem scheme shown in Fig. 1A in which we exploit the level alignment of two dyes to obtain a higher open-circuit voltage. One of the dyes should be a dye with a low lying HOMO (dye 1) and the second should have a LUMO aligned with the conduction band of TiO_2_ (dye 2). To optimize the efficiency of a tandem cell it has been proposed to have one species with an optical gap of 1.0 eV and another with 1.9 eV.^42^ However, while this is true for semi-conductors which absorb most photons above the band gap, for dyes with limited absorption it may be better to use two dyes with optical gaps of 1.1 eV in agreement with the Shockley–Queisser limit.^43^ Having dyes fulfilling these requirements, the basic idea can be described in six simple steps: I → II: a photon excites an electron from the HOMO of dye 2 to an excited level. II → III: the excited electron on dye 2 is rapidly injected into the conduction band of the semi-conductor. III → IV: a second photon excites an electron from the HOMO of dye 1 to the LUMO of dye 1. IV → V: the excited electron on dye 1 tunnels to fill the hole on the HOMO of dye 2. V → VI: an electron from the redox mediator regenerates the dye by filling the hole on the HOMO of dye 1. VI → I: the electron in the conduction band of the semi-conductor is used for performing electric work after which it is transferred back to the electrolyte *via* the counter electrode as in standard DSSCs.

**Fig. 2:**
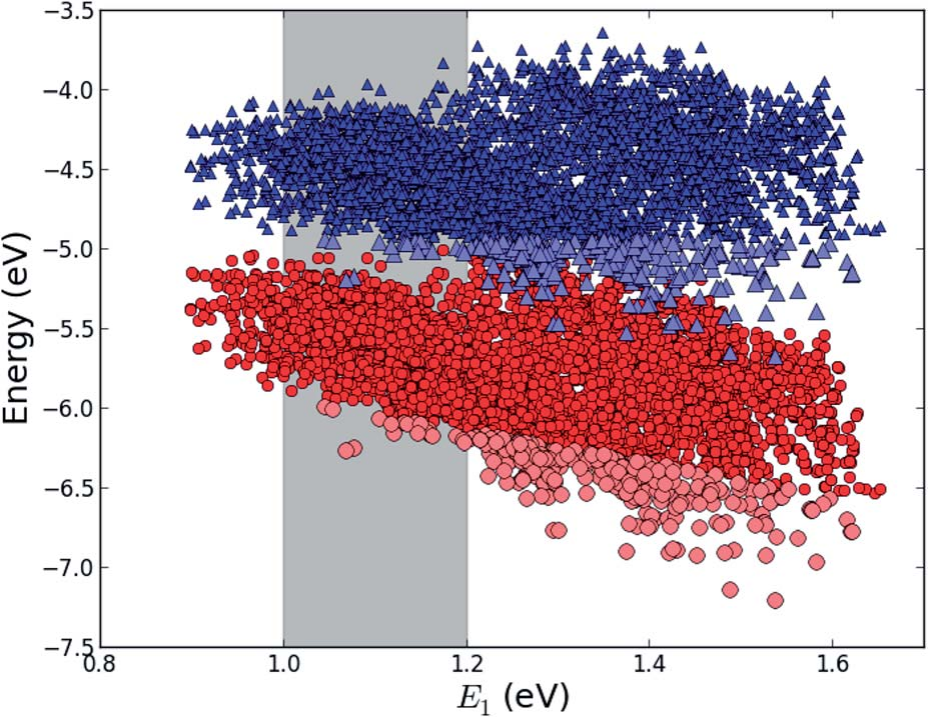
*E*_HOMO_ (red circles) and *E*_HOMO_ + *E*_1_ (blue triangles) for all 5000+ porphyrins in our database^22,23^ plotted against the lowest optical transition energy, *E*_1_. The light blue and light red points indicate dyes where *E*_HOMO_ + *E*_1_ lies at a lower energy than the maximum value of *E*_HOMO_ making it a potential candidate for dye 1 in the tandem scheme and the gray shaded area indicates the region of interest around *E*_1_ = 1.1 eV.

**Fig. 3:**
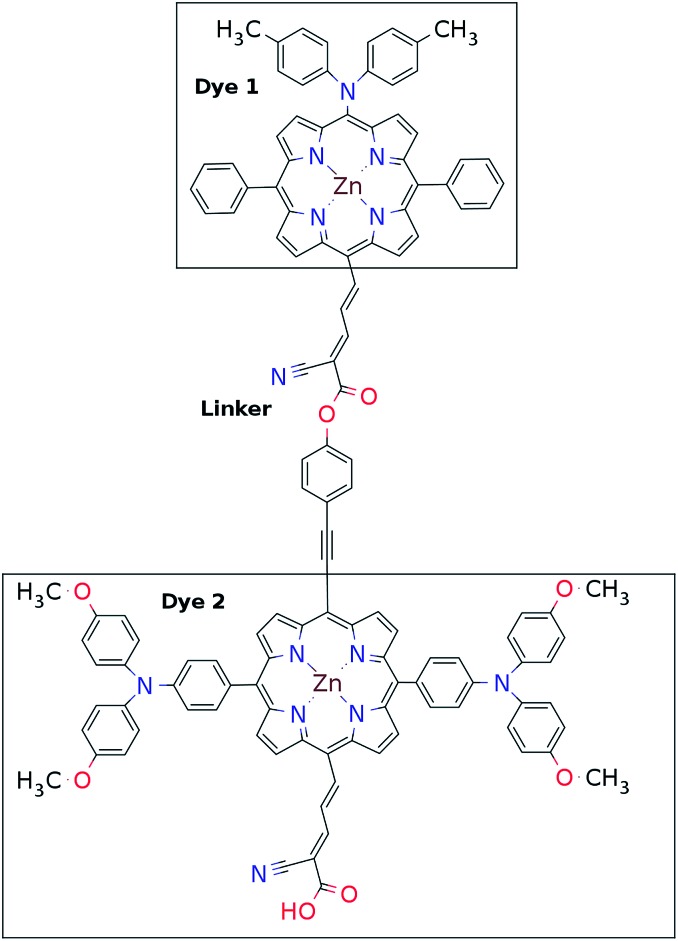
Chemical structure of one of the molecular tandem schemes used in the present work.

This mechanism puts some constraints on the two dyes to be used, but using our database of functionalized porphyrins,^22,23^ we have identified around 800 suitable dye pairs (see Fig. 2 for an illustration of this process) made from 9 unique dyes for use as dye 2, all functionalized with highly donating side groups. On the other hand, the dyes suitable for use as dye 1 should have less donating side groups in order to have a lower-lying HOMO. From the suitable candidates we have chosen the simplest example and refined the structure to provide an experimentally realizable molecule while retaining the alignment of the molecular levels. The singlet excitation energies and level alignment of the refined individual dyes are given in Table 1. It may be noted that the calculated singlet excitation energies agree within 0.2–0.4 eV to the excitation energies obtained using TD-DFT with B3LYP (see Table S1 in ESI†). To create the tandem scheme, the dyes have been connected from the central side group of dye 2 to the anchor group of dye 1. A scheme of the full tandem scheme is given in Fig. 3. As in a semiconductor tandem cell, the connection between the two dye molecules has to act as a diode in order to suppress recombination of the final electron with the initial hole.^44–46^ Any molecular wire can act as diode, as long as it is asymmetric, *i.e.*, the energy levels at the two ends of the wire are different. Many such molecular wires have been investigated, for example by *I*(*V*) spectroscopy of break junctions bridged by a molecule or of a molecule connecting the tip of a scanning tunneling microscope to a surface.^47–49^ The optimal length of the linker involves a trade-off between fast electron transfer (shorter is better) and preservation of the properties of the individual dyes (longer is better). A useful tool for controlling the charge transfer between the dye molecules is a tunnel junction, which contains a stretch of molecular wire with a significant HOMO–LUMO gap between conducting pi-systems.^50,51^ Macroscopic tunnel junctions constitute an important part of inorganic tandem solar cells. To minimize hybridization between the two dyes while ensuring a reasonably short tunneling barrier, we propose to connect the two dyes by an ester bond between the carboxylic acid of dye 1 and a phenolic group linked to the *meso* position of dye 2. The synthesis of the tandem structure is an arduous but realizable task. Two individual face-to-face functionalized porphyrins have to be prepared, showing a similar functionalization pattern to that of push–pull porphyrin dyes with record efficiencies in DSSCs.^25^ Each of the crosswise-substituted porphyrins can be obtained by condensation between dipyrromethane and either benzaldehyde (for dye 1) or *N*,*N*-bis(4-methoxyphenyl)-4-aminobenzaldehyde (for dye 2). Following this, bromination of the free *meso* positions of the porphyrins, and sequential Pd-catalyzed cross-coupling reactions, namely, Buchwald, Suzuki or Stille procedures, to incorporate the diphenylamino moiety, 4-ethynylphenol or the anchoring group, respectively, would lead to the target dyes. Finally, both chromophores could be linked together through a final esterification reaction.

**Table 1:** Calculated singlet excitation energies and level alignment of the individual dyes used in the tandem scheme

Species	*E*_HOMO_ (eV)	*E*_HOMO_ + *E*_1_ (eV)	*E*_1_ (eV)
Dye 1	–5.9	–4.7	1.2
Dye 2	–5.3	–4.1	1.2

**Fig. 4:**
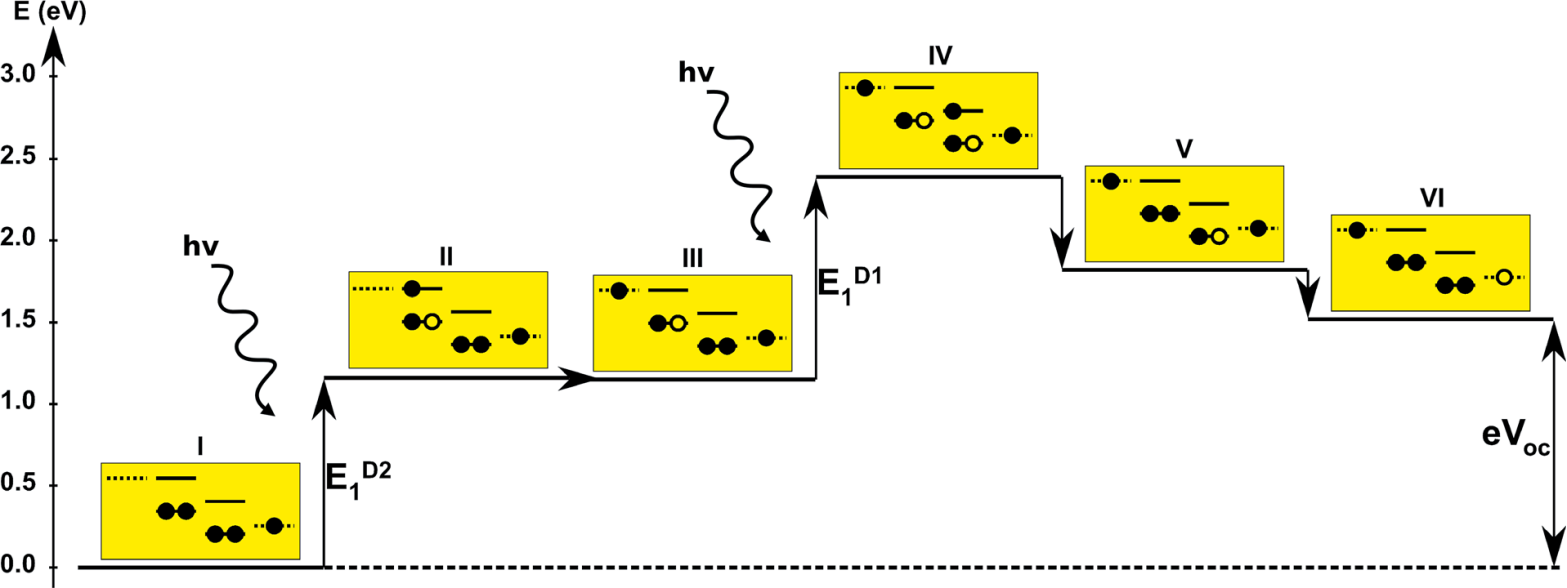
One-particle state-based sketch of the steps involved in the molecular tandem device proposed here. For each state the energetics are indicated by the *y*-axis while the one-particle mechanism associated with the step is shown in the corresponding yellow box. Here the filled circles represent electrons and the empty circles represents holes. From right to left in each box the dotted state is the redox mediator, the first set of solid lines represents the ground state and excited state of dye 1, the second set of solid lines represents the ground and excited state of dye 2 and the last dotted line represents the conduction band of the semi-conductor.

Proof of the lack of hybridization between dyes has been achieved by calculations of the frontier orbitals of the full tandem set up. A visualization of these using, respectively, the PBE and CAM-B3LYP functional are given in Fig. S1 and S2 in the ESI.† It may be noted that the different functionals yield different ordering and spatial weights, but in both figures it is readily seen that the orbitals are highly localized on the individual dyes and resemble the orbitals calculated for these individually. This means that the proposed mechanism effectively transfers an electron from the backbone of dye 1 to the anchor group of dye 2. In this way the tandem scheme achieves excellent charge separation with the electron overlapping with the conduction band of the semi-conductor and the hole located close to the electrolyte. Performing a TD-DFT calculation on the full tandem dyad furthermore reveals a large oscillator strength (see Table S2 in the ESI†) for the local excitations on both parts of the dyad. Additionally, the oscillator strength associated with the charge transfer from dye 2 to dye 1 in the dyad is of comparable size to the local excitations. Thus the charge transfer is possible at least in terms of energetics but the charge transfer integral has yet to be evaluated.^52,53^ Using the values from Table 1, and taking TiO_2_ as the semi-conductor with the conduction band located at –4.1 eV and an electrolyte with a redox potential aligned 0.3 eV above the HOMO of dye 1, we can construct the detailed energetics given in Fig. 4 where the mechanisms for all six steps are also indicated. In the figure we further assume that the electronic excitations are faster than the structural relaxation of the individual dyes. This leaves us with a mechanism that, apart from the two photo-excitations, is downhill and with a theoretical open-circuit voltage of 1.5 V which is a significant improvement compared to current DSSC devices. However, the low-lying [Co^II/III^(bpy–pz)_2_] redox pair only has a redox potential of –5.36 eV *vs.* vacuum^54^ which limits the theoretical open-circuit voltage of the tandem device to 1.26 V (using *V*_oc_ = *E*_c_ – *E*_red_). Thus, it is crucial to use an electrolyte with a lower redox potential. This could *e.g.* be achieved by modifying the ligands of the popular cobalt-based redox couple as shown by Feldt *et al.*^55^ or by designing completely new redox mediators.

### 3.2 Intermediate band scheme

In general the lifetimes of the singlet excitation of zinc porphyrins are long (more than 1 ns).^56,57^ However, the time-scale on which the excited electron on dye 1 tunnels to the hole on dye 2 is unknown (step IV → V in Fig. 4). Unfortunately, this crucial step is probably highly unlikely, since there are very few photoexcited electrons in the LUMO of dye 1, which have to find one of the very few photo-generated holes which are in the HOMO of dye 2 at the same place and the same time. A possibility to overcome this is to have the HOMO of dye 2 half-filled in the ground state as shown in Fig. 1B as this would give the photoexcited electrons a good chance to find a hole in the HOMO of dye 2. Having this type of scheme would then give the six step shown in Fig. S3 in the ESI.† I → II: a photon excites an electron from the HOMO of dye 1 to the LUMO of dye 1. II → III: the excited electron on dye 1 tunnels to the singly-occupied HOMO of dye 2. III → IV: a second photon excites an electron from the now doubly-occupied HOMO of dye 2 to an exited state on dye 2. IV → V: the excited electron on dye 2 is rapidly injected into the conduction band of the semi-conductor. V → VI: an electron from the redox-mediator regenerates the dye by filling the hole on the HOMO of dye 1. VI → I: the electron in the conduction band of the semi-conductor is used for performing electric work after which it is transferred back to the electrolyte *via* the counter electrode as in standard DSSCs.

A possibility for realizing this scheme could be to use a porphyrin with a transition metal center with an uneven number of electrons such as Fe^3+^ (d^5^-system). Performing a calculation of this species confirms in accordance with reported calculations,^58,59^ the presence of an unpaired electron located in an orbital similar to the HOMO of the iron(ii) porphyrin (see Fig. S4 in ESI† for details).

### 3.3 Intermediate triplet state scheme

**Fig. 5:**
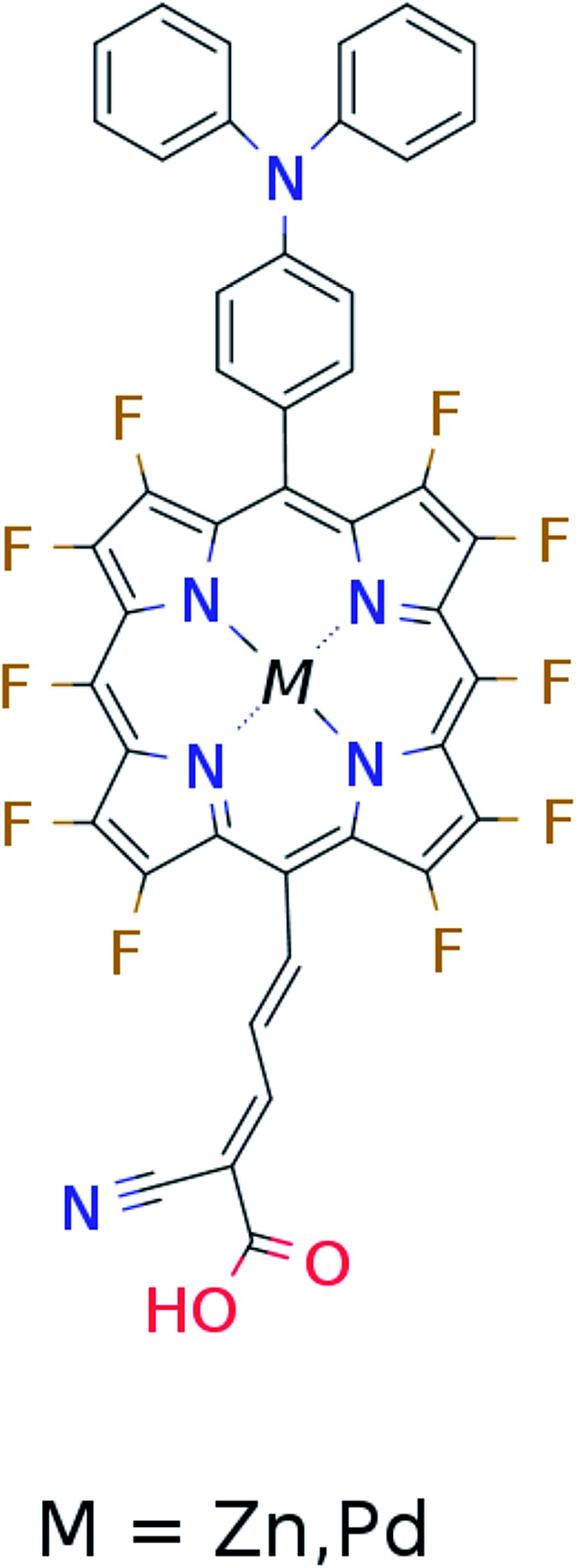
Scheme of the proposed dye for the single-dye intermediate triplet state scheme.

**Fig. 6:**
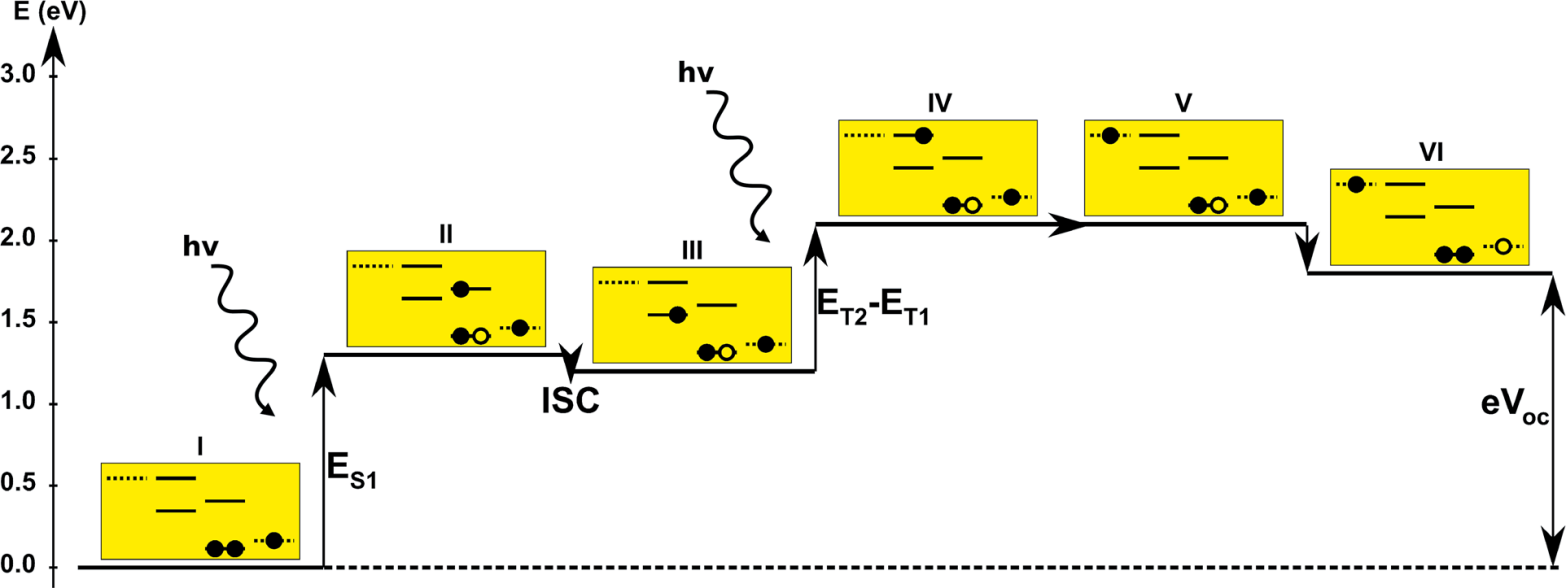
One-particle state-based sketch of the steps involved in the single-dye intermediate triplet state scheme. For each state the energetics are indicated by the *y*-axis while the one-particle mechanism associated with the step is shown in the corresponding yellow box. Here the filled circles represents electrons and the empty circles represents holes. From right to left in each box the dotted state is the redox mediator, the first set of solid lines represents the singlet ground state and the first singlet excited state of the dye, the second set of solid lines represents the first and second triplet excited state of the dye and the last dotted line represents the conduction band of the semi-conductor. The step involving inter-system-crossing is marked by ISC.

In the single-dye intermediate triplet state scheme we propose to still use two photons but only a single dye. Here the idea is to have a dye with a low lying HOMO, a LUMO located inside the gap of the semi-conductor used and a higher excited state aligned with the semi-conductor conduction band edge. The first photon should thus excite an electron from the HOMO to the LUMO followed by a second photon exciting the electron from the LUMO to the higher excited state. To ensure that the first excitation lives long enough for the second excitation to occur, we can exploit inter-system-crossing (ISC) to prepare the first excited state in a triplet state with a long lifetime. This is equivalent to the scheme in Fig. 1C. Thus, the idea can be described in six steps: I → II: a photon excites an electron from the HOMO of the dye to the first excited singlet state. II → III: the excited electron undergoes ISC to the first excited triplet state. III → IV: a second photon excites the electron from the first excited triplet state to a higher excited triplet state. IV → V: the excited electron is injected into the conduction band of the semi-conductor. V → VI: an electron from the redox-mediator regenerates the dye by filling the hole on the HOMO of the dye. VI → I: the electron in the conduction band of the semi-conductor is used for performing electric work after which it is transferred back to the electrolyte *via* the counter electrode as in standard DSSCs. By again employing our database,^22,23^ we have found a candidate (see Fig. 5) with suitable energy levels as shown in Table 2. Here, the LUMO is located in the band gap of TiO_2_ and the triplet is located at a slightly lower energy than the singlet state making it energetically favorable to perform ISC. Furthermore, the triplet LUMO+2 is well aligned with the conduction band of TiO_2_ making this level perfect as the second excited state used in this type of scheme. Using these levels in the dye yields the energetics shown in Fig. 6 where the mechanisms for all six steps are also indicated. From the figure it is seen that this scheme yields a *V*_oc_ around 1.8 V, again significantly exceeding 1.0 V. However, for this approach to be realistic we need a dye with a high ISC. As the fluorescence lifetime of a zinc porphyrin molecule has been reported to be greater than 1.0 ns, indicating no significant ISC,^56^ we may need to exchange the Zn metal center with a heavier metal to obtain a higher ISC yield. Here, porphyrins especially with a Pd metal center have previously been shown to undergo efficient ISC with a quantum yield close to unity.^60^ Performing a DFT calculation on the closed-shell Pd version of the porphyrin dye reveals that the KS eigenvalues for the frontier orbitals are nearly identical to the eigenvalues for the Zn porphyrin. All eigenvalues are given in Table S3 in the ESI.† A visualization of the relevant orbitals for both species is given in Fig. S5 in the ESI.† From the figure we see that the orbitals are nearly identical for the two species and that the orbitals are very well suited for this type of scheme as the HOMO is located mostly on the backbone and side group whereas the LUMO is located more on the anchor group. This has the consequence that the first excitation obtains a great charge separation limiting the recombination of hole and electron. The shape of the second excited state (LUMO+2) of both porphyrins is very similar to the LUMO yielding a high oscillator strength for the transition. The localization of the LUMO+2 on the anchor group furthermore ensures a very fast injection of the excited electron into the conduction band of the semi-conductor. Using the Pd species may thus, *via* the high ISC, be a way to obtain the mechanism sketched in Fig. 6. Another approach to obtain the triplet excited state could be to use the concept of singlet fission.^61^ To use singlet fission we would however need to meet a number of restrictions such as a large energy gap between the singlet and triplet states, which is not present for the first excited state of the Zn porphyrin and a different type of dye should thus be used. As for the tandem scheme, the redox potential of the commonly-used redox mediators are located at energies too high for this scheme to be efficient. Thus, it is also crucial here to use an electrolyte with a better-aligned redox potential.

**Table 2:** Calculated singlet and triplet orbital energies for the dye used in the single-dye intermediate triplet state scheme

State	*E*_singlet state_ (eV)	*E*_triplet state_ (eV)
HOMO	–6.2	—
LUMO	–4.9	–5.0
LUMO+2	—	–4.1

## 4 Conclusions

We have proposed three new two-photon tandem schemes for use in photovoltaics in a pure molecular framework thus avoiding the inherent problems of disorder and defects found for solid state photovoltaics. In all cases, high energy electron–hole pairs are generated by absorption of two photons which allow for higher output voltages while harvesting a broader range of the solar spectrum. The three considered schemes include a two-dye tandem structure, a two-dye intermediate band scheme, and a single-dye intermediate triplet state scheme. For all three schemes, we used an extensive database of porphyrin orbital energies to identify dyes with properly-aligned energy levels to yield open-circuit voltages well beyond 1.0 V. The proposed schemes were substantiated by *ab initio* calculations for the complexes indicating that the energy level alignment is retained upon attaching the molecules *via* a diode. Many possible loss mechanisms and questions regarding *e.g.* the synthesis of the complexes, the efficiency of electron transfer between the dyes, the life times of the generated electron–hole pairs, *etc.* are discussed, and possible improvements by means of modifications to the molecules are suggested. The present work has established a new concept of molecular tandem-based devices that could have important implications in photovoltaic applications. Work towards a proof of concept based on the results of this work is being conducted.

## Supplementary Material

Supplementary information
