# Supplementary material for: Design of two-photon molecular tandem architectures for solar cells by ab initio theory
Source: Chem Sci. 2015 Mar 4;6(5):3018–25. doi: 10.1039/c4sc03835e (PMC5657411; doi:10.1039/c4sc03835e)
Supplement: Supplementary file 1 [file SC-006-C4SC03835E-s001.pdf]

Electronic Supplementary Material (ESI) for Chemical Science.
This journal is © The Royal Society of Chemistry 2015

Supporting Information for Design of two-photon molecular tandem architectures for solar cells by *ab initio* theory

Kristian B. Ørnsø, Juan M. Garcia-Lastra, Gema De La Torre, F. J. Himpsel, Angel Rubio and Kristian S. Thygesen

Additional computational details

Amsterdam Density Functional[1] (ADF, version 2012) calculations were carried out using the all-electron basis of DZP quality (double-ζ Slater-type orbitals plus one polarization function) given in the program database. The precision numerical integration parameter was set to 5.0. The mixing parameter during the Self-Consistent Field procedure was set to 0.1. Atomic positions were relaxed until the default convergence thresholds of the code were reached. For the sake of consistency with the GPAW calculations, PBE[2] functional was used to describe the exchange-correlation energy. In the same vein, both, singlet and triplet excitation energies were computed following the same ΔSCF procedure as in the GPAW calculations.

Orca[3] TD-DFT calculations on the molecular tandem scheme were performed with the CAM-B3LYP[4] functional and the def2-TZV[5;6] basis set with the Stuttgart-Dresden SD(2,MWB)[7] Effective Core Potential (ECP) for C, N and O and SD(10,MWB)[8] for Zn. The ECPs were used due to the size of the dyad. For the calculation standard convergence criterion were used and a total of 10 excitations were considered.

Table S1: Excitation energies for the individual dye 1 and 2 calculated using TD-DFT with PBE and B3LYP as implemented in ADF

Dye	PBE E_1 (eV)	B3LYP E_1 (eV)
Dye 1	1.00	1.60
Dye 2	0.56	1.40

Table S2: Major orbital contribution and oscillator strength for the 3 most important excitations in the proposed molecular tandem scheme dyad calculated using TD-DFT in Orca with the CAM-B3LYP functional

Major orbital contribution	f_{osc}
HOMO-1 $\rightarrow$ LUMO	1.47
HOMO $\rightarrow$ LUMO+1	0.17
HOMO $\rightarrow$ LUMO	0.11

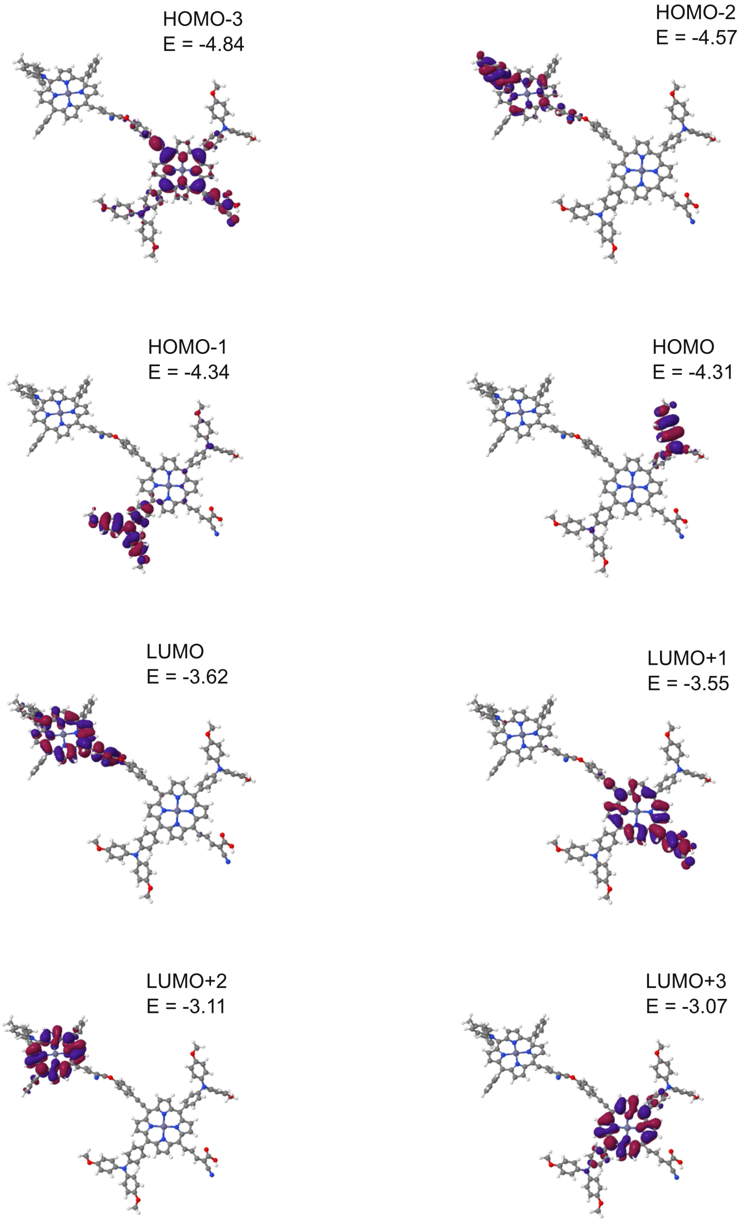

Figure S1: Visualization of the frontier orbitals in the tandem setup calculated with PBE. The HOMO and LUMO+1 of the full setup correspond to the HOMO and LUMO of dye 2 while the HOMO-2 and LUMO correspond to the HOMO and LUMO of dye 1. Kohn-Sham eigenvalues in eV are indicated in the figure. The figures are created from cube files created with GPAW and visualized with Jmol.[9] The isosurfaces are created with a cutoff of 0.05 (blue) and -0.05 (red).

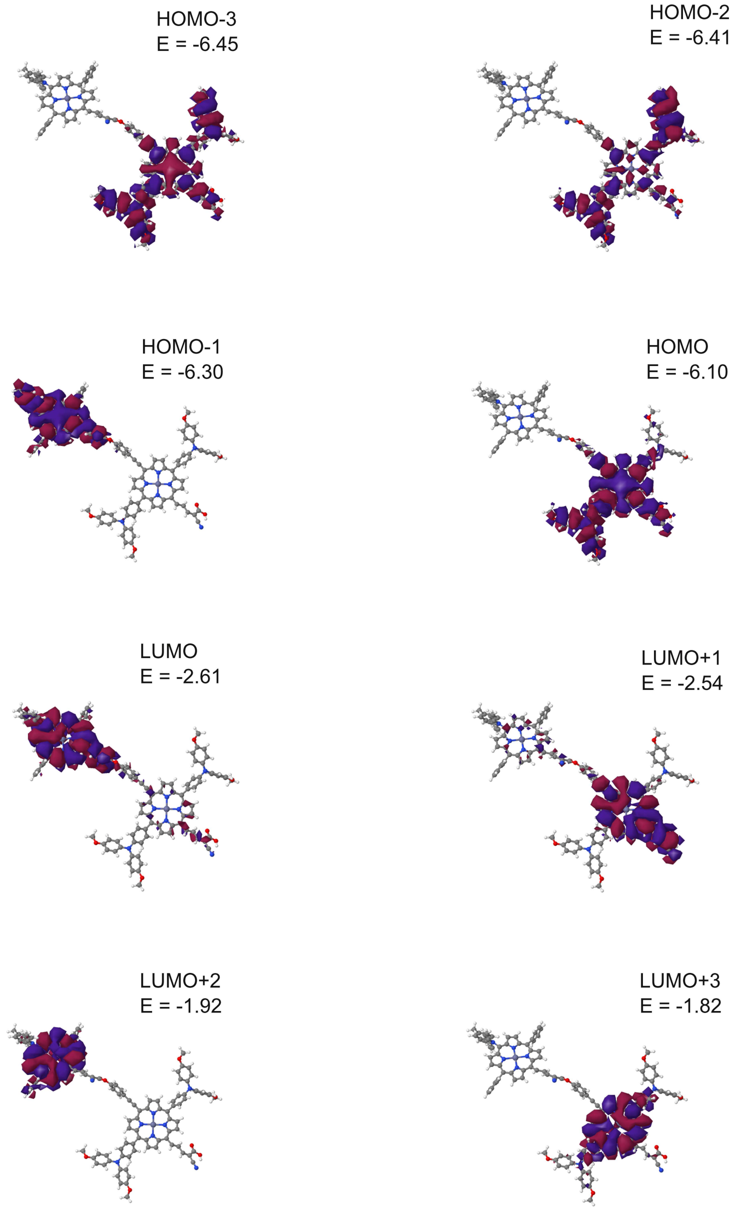

Figure S2: Visualization of the frontier orbitals in the tandem setup calculated with CAM-B3LYP. The HOMO and LUMO+1 of the full setup correspond to the HOMO and LUMO of dye 2 while the HOMO-1 and LUMO correspond to the HOMO and LUMO of dye 1. Kohn-Sham eigenvalues in eV are indicated in the figure. The figures are created from cube files created with Orca and visualized with Jmol.[9] The isosurfaces are created with a cutoff of 0.005 (blue) and -0.005 (red).

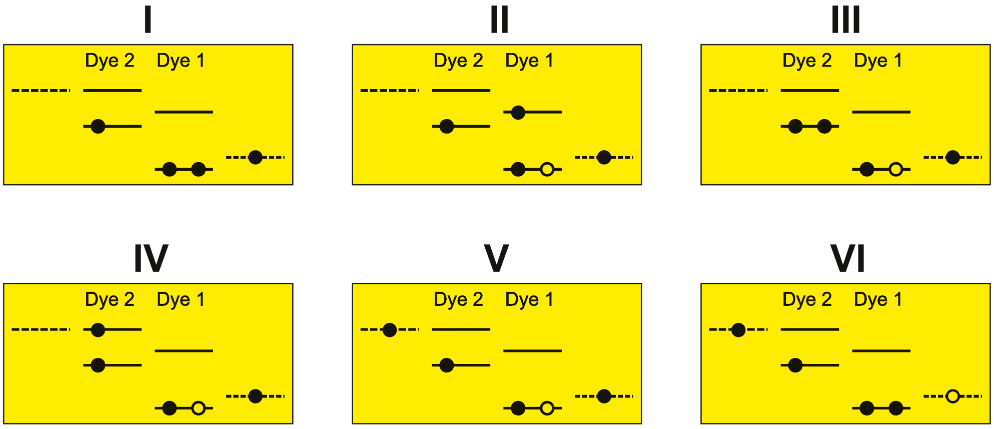

Figure S3: One-particle state based sketch of the steps involved in the intermediate band scheme. Here the filled circles represent electrons and the empty circles represents holes. From right to left in each box the dotted state is the redox mediator, the first set of solid lines represents the ground state and excited state of dye 1, the second set of solid lines represents the ground and excited state of dye 2 and the last dotted line represents the conduction band of the semi-conductor.

Table S3: Kohn-Sham eigenvalues for the Pd and Zn porphyrins proposed for the molecular interband setup

State	$E_{\mathrm{KS}}(\mathrm{Pd})$ (eV)	$E_{\mathrm{KS}}(\mathrm{Zn})$ (eV)
HOMO	-4.76	-4.76
LUMO	-3.99	-3.99
LUMO+1	-3.55	-3.56
LUMO+2	-3.43	-3.32
LUMO+3	-3.35	-2.25

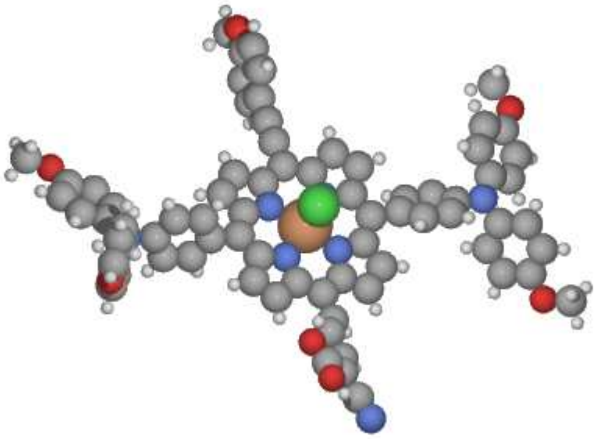

Figure S4: Illustration of the iron(III)chloride version of dye 2. The calculation has been performed using GPAW with the PBE functional as described in the Methods section with the exception that the magnetic moment has been fixed to create a doublet configuration.

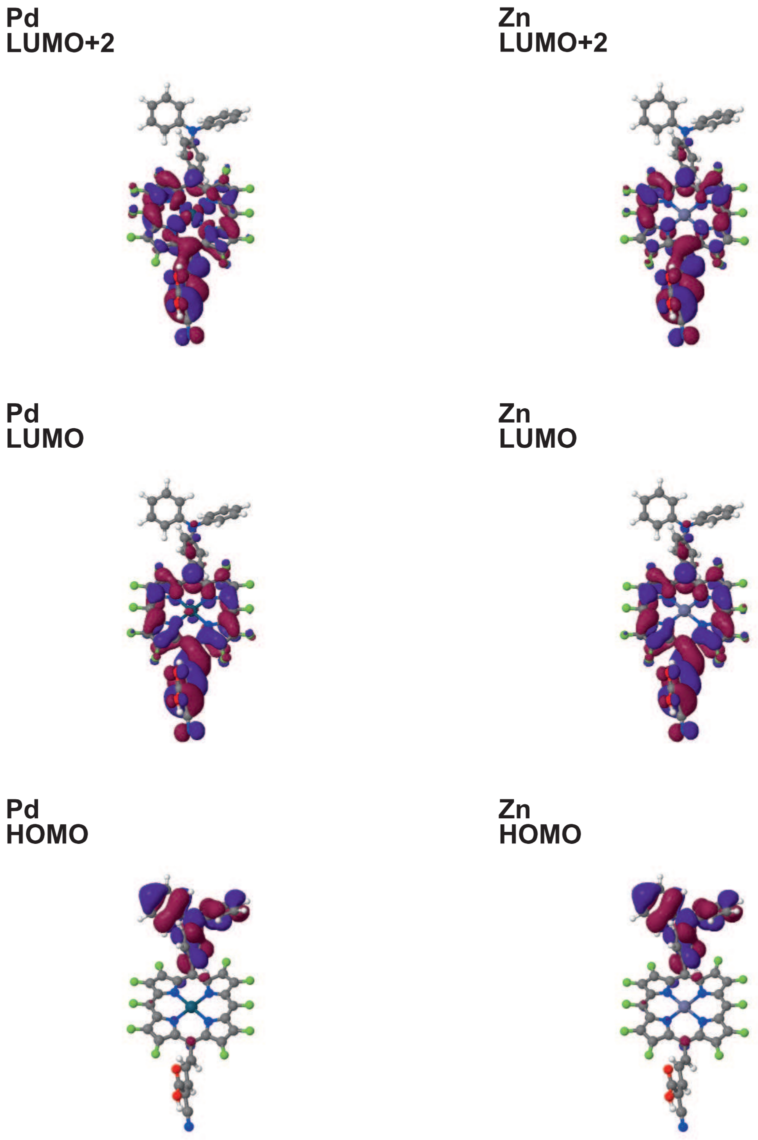

Figure S5: Visualization of the proposed active orbitals for **left:** the Pd version and **right:** the Zn version of the dye used in the single dye intermediate triplet state setup. The figures are created from cube files created with GPAW and visualized with Jmol.[9] The isosurfaces are created with a cutoff of 0.05 (blue) and -0.05 (red).

References

[1] G. te Velde, F. M. Bickelhaupt, E. J. Baerends, C. Fonseca Guerra, S. J. A. van Gisbergen, J. G. Snijders and T. Ziegler, *J. Comput. Chem.*, 2001, **22**, 931–967.

[2] J. P. Perdew, K. Burke and M. Ernzerhof, *Phys. Rev. Lett.*, 1996, **77**, 3865–3868.

[3] F. Neese, *Orca, Program Version 3.0.1*.

[4] T. Yanai, D. P. Tew and N. C. Handy, *Chem. Phys. Lett.*, 2004, **393**, 51–57.

[5] A. Schafer, H. Horn and R. Ahlrichs, *J. Chem. Phys.*, 1992, **97**, 2571–2577.

[6] F. Weigend and R. Ahlrichs, *Phys. Chem. Chem. Phys.*, 2005, **7**, 3297–3305.

[7] A. Bergner, M. Dolg, W. Küchle, H. Stoll and H. Preuss, *Mol. Phys.*, 1993, **80**, 1431–1441.

[8] M. Dolg, U. Wedig, H. Stoll and H. Preuss, *J. Chem. Phys.*, 1987, **86**, 866–872.

[9] *Jmol: an open-source Java viewer for chemical structures in 3D. http://www.jmol.org/*.
